# Health costs of reproduction are minimal despite high fertility, mortality and subsistence lifestyle

**DOI:** 10.1038/srep30056

**Published:** 2016-07-20

**Authors:** Michael Gurven, Megan Costa, Ben Trumble, Jonathan Stieglitz, Bret Beheim, Daniel Eid Rodriguez, Paul L. Hooper, Hillard Kaplan

**Affiliations:** 1 Department of Anthropology, University of California-Santa Barbara, Santa Barbara, CA, USA; 2 Population Studies Center, Graduate Group in Demography, University of Pennsylvania, Philadelphia, PA, USA; 3 Institute for Advanced Study in Toulouse, Toulouse, France; 4 Department of Anthropology, University of New Mexico, Albuquerque, NM, USA; 5 Department of Medicine, Universidad de San Simón, Cochabamba, Bolivia; 6 Department of Anthropology, Emory University, Atlanta, GA, USA.

## Abstract

Women exhibit greater morbidity than men despite higher life expectancy. An evolutionary life history framework predicts that energy invested in reproduction trades-off against investments in maintenance and survival. Direct costs of reproduction may therefore contribute to higher morbidity, especially for women given their greater direct energetic contributions to reproduction. We explore multiple indicators of somatic condition among Tsimane forager-horticulturalist women (Total Fertility Rate = 9.1; n =  592 aged 15–44 years, n = 277 aged 45+). We test whether cumulative live births and the pace of reproduction are associated with nutritional status and immune function using longitudinal data spanning 10 years. Higher parity and faster reproductive pace are associated with lower nutritional status (indicated by weight, body mass index, body fat) in a cross-section, but longitudinal analyses show improvements in women’s nutritional status with age. Biomarkers of immune function and anemia vary little with parity or pace of reproduction. Our findings demonstrate that even under energy-limited and infectious conditions, women are buffered from the potential depleting effects of rapid reproduction and compound offspring dependency characteristic of human life histories.

An evolutionary life history framework posits that in all organisms energy is limited and allocated to competing physiological demands in ways that optimize biological fitness. Natural selection often prioritizes maximizing earlier reproduction over longevity, so organisms may increase fertility at the expense of longevity. Under a wide range of conditions, it is expected that energetic resources invested in reproduction should therefore trade-off against resources invested in somatic maintenance^1^. Kirkwood’s “disposable soma” theory focuses on trade-offs to highlight the conflict between investments in somatic maintenance and reproduction^2^, leading to an accumulation of damage, faster senescence and lower post-reproductive survival, especially when exogenous mortality is high^3^,^4^. However, comparative tests in mammals and birds are inconclusive^5^. While several studies provide support for an inverse relationship between fertility and longevity in humans^6^,^7^, others show no, or even a positive, relationship between completed fertility and human lifespan^8^,^9^. It has been suggested that late life reproduction or late menopause may be positively associated with post-reproductive survival in humans^10^. However, many of these studies rely on historical demographic data, focus primarily on mortality, and are unable to control for differences in reproductive timing, maternal energy budgets and selection effects^11^,^12^.

Even if total reproduction is not consistently associated with lifespan as predicted by the original disposable soma hypothesis, the timing and intensity of reproduction can still generate costs that affect different aspects of maternal health and fitness. A relevant literature on “maternal depletion syndrome” examines trade-offs between fertility and health by studying both the short-term and cumulative long-term impacts of repeated pregnancies in developing countries. The additional nutritional and metabolic costs of pregnancy and lactation^13^, combined with immune modulation and increased susceptibility to certain infections during pregnancy^14^ can place a substantial burden on maternal health and nutritional status, at least in the short-term. For example, pregnancy in well-nourished women with gestational weight gain of 12 kg is estimated to cost an additional ~70 kcals/day in the first trimester to ~440 kcals/day in the third trimester^13^. Lactation is even more costly, increasing metabolic rate by ~25%, protein needs by up to 54% and vitamin/mineral needs up to 93%^15^. In the 1960’s, maternal depletion was identified as an important deterrent to women’s health in energy-limited populations with intensive breastfeeding patterns, based on research in rural Papua New Guinea showing an association between short inter-birth intervals and compromised nutritional status assessed using anthropometrics^16^. In the 1980’s and 1990’s, there were numerous attempts to test whether maternal depletion due to repeated, closely spaced bouts of reproduction negatively impacted maternal nutritional status. The evidence was largely mixed^17^, however, most studies to date use fairly small sample sizes, rarely examine both pre- and post-menopausal women, are cross-sectional thereby limiting causal inference. These studies often rely on one or two anthropometric measures (e.g. weight, body fat) to assess overall nutritional status, and overlook other aspects of somatic condition such as immune function, thus precluding more comprehensive analyses of maternal condition over the life course. Most studies also focus only on the period covering one or two births rather than the cumulative effects of multiple reproductive bouts. Another difficulty with observational studies is that the problem of phenotypic correlation confounds evidence of trade-offs; women in better condition and with greater resource availability may experience higher and faster fertility, and better health outcomes^1^,^18^.

Evidence suggests that short interbirth intervals (<18 months) are more harmful to maternal health than longer intervals, even though shorter intervals usually imply shorter lactation duration, due in part to higher overall daily energetic costs^19^. Short interbirth intervals are more likely to lead to maternal depletion when coupled with intensive breastfeeding and an inability for women to recover micro- and macronutrient stores (e.g. iron, vitamin A, folate, calcium) before investing in the next pregnancy^20^,^21^. Short interbirth intervals have also been linked to greater likelihood of puerperal endometritis, premature rupture of membranes, anemia and maternal death in a large urban Latin American sample^22^, but generalization is difficult due to a dearth of comparative studies^23^. Short interbirth intervals are also associated with worse infant outcomes, including preterm birth, low birthweight and neonatal death^24^. While long interbirth intervals (>5 years) are also sometimes associated with adverse maternal health outcomes such as preeclampsia^19^,^23^, these effects may be difficult to separate from other correlates of infecundity, poor maternal condition, and partner loss.

Rapid reproduction, multiple dependency and relatively high fertility of humans – greater than expected for a primate of our size - have all been highlighted as hallmark features of the evolved human life history^25^. Yet longitudinal studies of potential somatic costs to women in natural fertility societies are scant. Prior to widespread transitions to low fertility in industrialized populations the natural fertility profile was characterized by repeated pregnancies, intensive breastfeeding, and multiple concurrent dependent offspring. Despite the ubiquity of natural fertility throughout human history, whether cumulative reproductive effort and fertility timing affect somatic condition of women under these conditions, especially with minimal access to healthcare and labor-saving technology, is an important question that does not yet have a comprehensive answer.

Here, we test whether the intensity of female reproduction is associated with differential nutritional and immunological health in adulthood among the Tsimane, a Bolivian natural fertility population of forager-horticulturalists. We test among pre- and post-menopausal women whether cumulative numbers of live births (parity) and the timing between births (pace) predict several dimensions of maternal health, independent of age and other potential confounders like schooling and Spanish fluency, which act as proxies for access to markets and the nearest town. Measures of health include anthropometric indicators of nutritional status, including weight and body fat percentage. We also include hemoglobin as a biomarker of anemia to assess iron status, white blood cell count (WBC) as a measure of immune activation due to infection, and erythrocyte sedimentation rate (ESR) as an additional immune biomarker of non-specific inflammation. Causes of high ESR include anemia of acute or chronic disease, macrocytosis, rheumatoid arthritis, tuberculosis, other bacterial infections, and high fibrinogen^26^. If higher fertility and faster reproductive pace result in compromised immune defenses (potentially acting synergistically with nutritional deficits) and greater morbidity, we would expect parity-specific increases in WBC and ESR. This study thus represents the first direct test of trade-offs between reproduction and several aspects of somatic condition among women using longitudinal data from a contemporary natural fertility population living a subsistence lifestyle.

Tsimane are an ideal test population because fertility is high, (total fertility rate [TFR] = 9.1), birth spacing is short (mean IBI = 30 months), breastfeeding is intensive and on-demand (mean weaning age = 19 months), and effective birth control is rare^27^,^28^. Burden of infectious disease is high, physical work load is heavy, and women have short stature and minimal obesity due to energetic limitation (Table S1). Tsimane women show indicators of poor health across a variety of dimensions. For example, adult women age 20+ have low bone mineral density despite a relatively high physical activity level^21^,^29^, high prevalence of self-reported depression symptoms despite relatively low economic inequality between or within sexes^30^, and gynecological morbidity – including a nontrivial fraction from sexually transmitted infection – despite an early age of marriage and stable marriages^31^. Bone mineral density is lower among women with higher fertility and shorter birth spacing^21^. Women of reproductive age also have elevated WBC count (53% > 10.0 × 10^9^ cells/L, Table S1) and ESR (80% above 20 mm/hr, Table S1)^32^. Ongoing biomedical surveys show high rates of anemia (28.0%, Table S1) and microalbumuria (27.6% with Albumin-Creatinine Ratio ≥ 30 μg/mg) consistent with kidney dysfunction and/or urinary tract infection, perhaps as sequelae of high reproductive burden. By age 60, women have lost roughly 80% of their teeth, report significant pains affecting mobility in numerous joints and bones, and show evidence of functional limitations (e.g. loss of balance, greater pain while weaving, kyphosis)^33^. The proportion of women presenting with advanced (grade 3+) cystocele (bladder prolapse) is relatively high (9.7%), higher than that observed in age-matched U.S. women^34^.

## Study Goals

We first examine categorical longitudinal trajectories of anthropometric variables to test whether variation in parity and/or reproductive pace is associated with long-term decreases in nutritional status. We next employ multilevel (or mixed effects) regression models using longitudinal data to examine short-term changes in weight, body mass index (BMI), body fat percentage, hemoglobin (Hb), ESR and WBC as a function of parity and pace of reproduction among women aged 15–44 years (n = 592). Among women age 45+ (n = 277), we test whether parity and pace of reproduction are associated with the same health measures. This study reports associations between demographic, nutritional and immunological variables on the largest sample size to date of women living under conditions most similar to those of our preindustrial past.

## Results

### Trajectories of nutritional status and predictors of consistent anthropometric decline

**Figure 1:**
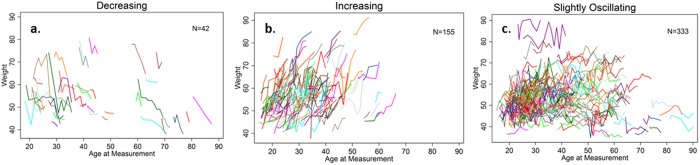
Longitudinal trajectories of weight among women age 15+. Women with (**a**) sustained weight decline, or oscillations with mean decrease >0.3 standard deviations (SD); (**b**) sustained weight gain, or oscillations with mean increase >0.3 SD; (**c**) oscillations with mean increase or decrease <0.3 SD.

We first categorize longitudinal trajectories of women’s anthropometric status to assess whether reproductive variables are associated with sustained declines in nutritional condition. Anthropometric trajectories of women based on repeated measures are displayed in Figs 1, S1 and S2, separated into three categories: women whose measurements on average (a) decrease over time, (b) are relatively stable (i.e. slightly oscillating within 0.3 SD of individual-level mean), and (c) increase over time. Roughly 7–9% of women were in the “decreasing” category, 26–29% in the “increasing” category, and 62–64% in the “slightly oscillating” category (37–40% of which were slightly decreasing) (Table 1). Neither parity nor pace of reproduction (1/IBI) is associated with the probability of having a decreasing nutritional status (weight, body fat or BMI) over time (Table 2). Younger and uneducated women were more likely to be in the declining body fat category. Time elapsed since last birth was also associated with declining body fat (Table 2). Adding interactions of covariates with parity or pace of reproduction does not improve model fit or yield significant parameter estimates (Table S2). Multinomial regressions predicting declining nutritional status relative to the other two categories show a similar lack of evidence that parity or reproductive pace are associated with poorer long-term nutritional status (Table S3).

### Changes in nutritional status over time

We separate analyses of parity and pace on short-term changes in health outcomes among reproductive aged women (ages 15–44) and those who have completed reproduction (age 45+). Mixed effects models (see *Materials: Analysis*) account for repeated measures over time, allowing woman-specific intercepts and slopes in health trajectories, while adjusting for time-varying covariates and fixed effects.

### Women Age 15–44

**Figure 2:**
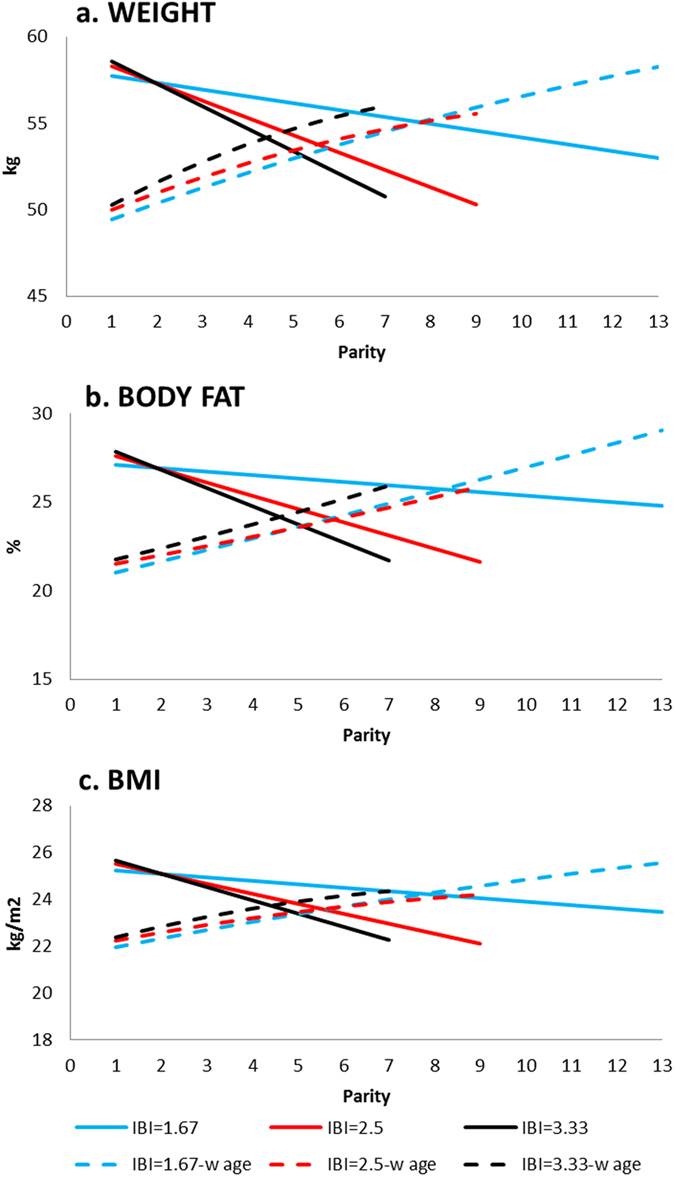
Effects of parity and inter-birth interval on women’s nutritional status for women aged 15–44. Panels include (**a**) weight, (**b**) body fat percentage, and (**c**) BMI. Interbirth interval (IBI) defined as the mean ± 1 SD (2.50 ± 0.83 years). Solid lines convey predicted marginal effect of parity and IBI, holding all other variables at sample averages, including age. Dashed lines use average age at first birth for parity = 1, and then add the IBI to each age in the regression models given in Table 3. Average age at first and last birth is 18 and 38, respectively.

In the full model with interactions (Table 3), main effects of parity and reproductive pace are both negatively associated with weight gain, body fat and BMI. The interaction of these two variables is positive, whereby faster pace among those with higher parity is associated with greater anthropometric status (higher BMI and body fat). The joint negative effects of parity and pace, combined with their positive interaction shows anthropometric decline with increasing parity, but those with slower pace (i.e. *longer* IBIs) show the steepest declines at higher parity (Fig. 2; Table 3). Holding age and other covariates constant, the cumulative effect of having nine births (the population TFR) versus none (the minimum value in the present sample) is 11.4 kgs lower body weight, 6.8% lower body fat, and 3.9 kg/m^2^ lower BMI, assuming an average IBI of 30 months. This represents 21% of the mean weight, 27% of mean body fat, and 16% of mean BMI for women age 15–44 (Table S1).

However, in most final models, nutritional status increases with age; controlling for other factors, aging one decade is associated with 10.5 kg weight, 4.5% body fat and 4.0 kg/m^2^ BMI gain in women 15–44, and 12.2 kg weight and 4.3 kg/m^2^ BMI gain in women age 45+ (Table 3). If we consider that a woman’s age must increase with parity over her lifetime, the model then predicts a different net result. The correlation between parity and age is strong, as would be expected for a natural fertility population (Pearson r = 0.80, p < 0.0001). The coefficients of age on weight, body fat and BMI in Table 3 are all positive, with slightly negative age^2^ terms. Incorporating age into the predictive model by starting from the age of first birth (18 years), and adding the IBI as an age increment for each successive child until the age of last birth (38 years) more than compensates for the negative impacts of parity and pace on nutritional status described above (compare dashed lines with solid lines in Fig. 2). The anthropometric gains as a function of only age added to the deficits from only parity and pace suggests that over a woman’s reproductive life of having nine births with a 30 month IBI (population mean), the average woman gains 5.6 kgs, 4.4% body fat, and 2.0 kg/m^2^ in BMI (Figs 2 and S3). A woman with a short IBI of 20 months will have 13 births, and gain on average 8.8 kgs, 8.0% body fat and 3.6 kg/m^2^ BMI, whereas a woman with a long IBI of 40 months will have 7 births and gain 5.7 kgs, 4.2% body fat and 2.0 kg/m^2^ (Figs 2 and S3). Results are robust to alternative models that limit data to women measured on at least two occasions (Table S8). Those who started reproducing at earlier ages were heavier, and had higher body fat percentage and BMI. Fluent Spanish speakers have significantly better nutritional status across all three measures (Table 3), but a marginally significant interaction term suggests less weight gain at higher parities among more fluent Spanish speakers (β_spanish*pace_ = −3.866, p = 0.084).

### Women Age 45+

Among older women whose reproduction is already complete, we find no evidence that higher parity or faster reproductive pace is associated with poorer nutritional status (Table 3). Adding interactions between reproductive variables and other indicators of maternal condition or human capital are not significant and do not improve model fits. Age is positively associated with weight and BMI, while Spanish fluency is positively associated with all three anthropometric measures. Results do not change when considering only women sampled at least two times (Table S9).

### Changes in health and survival: biomarkers of nutrition and immune function

Next we examine whether parity and reproductive pace are associated with changes in Hb, WBC count and ESR (Fig. S4). Model results for continuous variation in the biomarkers are given in Table 4, and those based on clinical cutoffs in Table S4.

### Women Age 15–44

In fully adjusted models, neither parity nor reproductive pace is significantly associated with either continuous variation in biomarkers (Table 4) or using clinical thresholds (e.g. anemia, leukocytosis, elevated ESR) (Table S4). Among pregnant women, odds of being anemic are almost 5 times greater, and odds of elevated ESR 2.6 times greater than among non-pregnant women. Best-fit models based on AIC included a measure of maternal energetic status (e.g. body fat percentage or weight). Only these indicators are associated with lower morbidity (i.e. higher hemoglobin and lower anemia, lower WBC count and leukocytosis, and lower ESR). Spanish fluency is associated with lower WBC count (and lower leukocytosis).

### Women Age 45+

Neither parity nor reproductive pace is significantly associated with hemoglobin (Table 4). However, faster reproductive pace is associated with higher WBC count, but the association is attenuated among women who are fluent in Spanish and who have higher body fat. Faster reproductive pace and higher body fat are associated with lower ESR, and there is a trend towards a positive interaction of parity and pace with ESR; the net impact of main effects and the interaction is increasing ESR with parity at short IBIs (Fig. S5).

### No evidence of mortality selection

Neither parity nor pace of reproduction predicts the probability that a woman died by the end of 2012, after controlling for age (Cox proportional hazards model, HR_parity_ = 1.035, p = 0.514; HR_1/IBI_ = 1.086, p = 0.785; HR_age_ = 1.068, p < 0.0001; n = 35 deaths (three deaths were within one year of a birth, and thus potentially could be associated with complications from childbirth)).

## Discussion

Among women of the same age during reproductive years, greater parity and faster reproduction are consistently associated with poorer anthropometric outcomes as measured by weight, BMI and body fat percentage, whereas parity and reproductive pace exert no substantial effects on biomarkers of nutrition and immune activation. Considering the joint effects from the full models, however, any pure deficits from repeated bouts of reproduction are offset by increasing age, as aging is consistently associated with gains in weight, body fat and BMI. Thus, within women there is no evidence of cumulative nutritional decline over the duration of multiple reproductive bouts over the lifetime. Post-reproductive women continue to gain weight, body fat, and BMI with age, irrespective of any short-term deficits during their reproductive years. Our findings thus appear to be inconsistent with simple trade-off models like the disposable soma hypothesis that posit inverse relationships between reproductive effort and health. Indeed, longitudinal trajectories display a low prevalence (7–9%) of sustained declines in nutritional status (Figs 1, S1 and S2). Consistent with these longitudinal findings in reproductive aged women, no robust, consistent harmful effects of cumulative, fast-paced reproduction were found in women who had already completed their reproduction. Unlike previous studies, these results were obtained from a large sample combining cross-sectional and longitudinal analyses with suitable time-varying and fixed effect controls that can isolate the association between reproductive parameters and potential maternal depletion. The mixed effects models controlled for confounding factors affecting short-term changes in nutritional status and health such as timing since the most recent birth and pregnancy status. Indeed, women sampled shortly after a birth had lower BMI and higher anemia, and women sampled during pregnancy were more anemic, had higher ESR, were heavier and had higher BMI. Other covariates expected to impact maternal condition (e.g., seasonality, schooling) are similarly adjusted in our models. Mortality selection also cannot explain the minimal effects of reproduction because neither parity nor pace of reproduction predicted the probability that a woman in this sample died by the end of the observation period.

One limitation is that our measures of parity and pace of reproduction are indirect measures of reproductive costs^35^. Daily energetic costs of lactation outweigh those of pregnancy^13^,^14^, but we do not have data on breastfeeding duration or intensity. However, all Tsimane women breastfeed their infants; Tsimane women breastfeed exclusively for about four months, then gradually introduce complementary foods, with weaning complete by 19–27 months^28^,^36^. Women commonly breastfeed up to and sometimes during early pregnancy. Our measure of reproductive pace, based on inter-birth intervals and accounting for time elapsed since the most recent birth, may indirectly adjust for variability in breastfeeding intensity among women, and our maternal condition variables may adjust for differences in energy budgets that might otherwise explain variability in the duration of postpartum lactational amenorrhea. Indeed, the steeper decline in anthropometric status we observed among women with longer IBIs (Fig. 2) is consistent with a longer duration of higher energetic lactational costs. However, it is also possible that our measures of maternal condition do not account for phenotypic correlation whereby more robust and healthier women can afford to reproduce at a faster rate but pay fewer costs than women who reproduce at a slower rate. Another limitation is the reliance on relatively crude measures of anthropometric status: weight, BMI and body fat percentage. Even though these are routinely used in clinical settings to assess chronic nutritional health and acute changes^37^, it is possible that greater reproductive costs lead to deficiencies in micronutrients such as iron, folate and zinc^20^,^23^,^38^. Here we show at least that cumulative reproductive burden does not impact anemic status; only pregnancy and having had a recent birth predicted higher risk of anemia (Table S4).

Overt maternal depletion with repeated reproduction may exist only under relatively extreme conditions of malnourishment and disease, as observed initially in Papua New Guinea in the 1960’s. Although energy-limited and often engaging in heavy physical labor^39^, Tsimane women on average are not malnourished in terms of overall caloric intake. The prevalence of underweight (BMI ≤ 18.5) among adult women is relatively low for women age 15–44 (1.2%) and age 45+ (3.9%). Similarly, the presence of obesity (BMI ≥30 kg/m^2^) was also low in ages 15–44 (5%), and age 45+ (9%) (Table S1). The high fertility and short IBIs are instead consistent with favorable economic and social strategies for coping in the otherwise harsh conditions of the Tsimane environment. Among Tsimane, horticulture provides a rich, fairly predictable and relatively easy-to-obtain source of carbohydrates, whereas fishing and hunting provide adequate protein and fat^40^.

It may also be the case that reproductive costs are difficult to detect because there is no unbiased control group of women who never reproduce; only 2% of women over age 45+ had never experienced a live birth in our demographic sample. Nulliparous women age 45+ tended to be shorter, lighter, have lower BMI, and more anemic than their age-matched parous counterparts (Table S5). A few of these women had secondary sterility from infection or ovarian cysts, and were likely to be either unmarried or to be a co-wife in a polygynous household. Thus, these women may have been in worse condition and/or may receive less “social security” from having no adult offspring who could potentially help feed them.

Although parity and pace were not associated with anemia, inflammation or leukocytosis, other conditions and health indicators may be more sensitive to the cumulative burden of fast reproduction. In other populations, for example, the likelihood of maternal and infant mortality increases with more births that are closely spaced^41^, and post-reproductive mortality is generally higher among nulliparous women and those with high parity^11^. Indeed, Tsimane female mortality exceeds male mortality the most (50% higher) during the reproductive period^42^. Over the period from 1972–2012, we document 30 maternal deaths from 4,275 live births, resulting in a maternal mortality rate of 702/100,000; this is higher than the rate in 94% of 183 countries in 2012^43^. Other indications suggest different costs of reproduction beyond mortality and nutritional status. Tsimane women exhibiting greater reproductive effort (higher parity, faster pace, earlier age at first birth) have lower calcaneal bone mineral density than their age and sex-matched peers after controlling for anthropometric status^21^. Lower bone mineral density is associated with increased risk of reported fractures in adulthood among Tsimane. Cystocele is another relatively important health problem observed in this population, and more prevalent than observed elsewhere^34^. Women report discomfort and painful urination, and cystocele may be partly responsible for repeated urinary tract and bladder infections, as well as for the microalbuminuria that is more common in Tsimane women than men. Lastly, a relatively unexplored possibility is that under certain conditions, women may maintain their soma at the expense of investment in the current, and possibly previous, offspring. Resource partitioning between mother and fetus will depend on maternal condition^44^. Consistent with this notion, Tsimane children of higher birth orders are more stunted and lower weight-for-age than those early in the birth order, especially for underweight mothers^45^. Understanding the conditions under which reproduction exerts specific costs on women’s welfare (and of their children) is critical for improved, personalized recommendations on birth spacing and maternal supplementation, and for helping improve maternal and child health.

That Tsimane women recoup most losses of nutritional status during and after reproduction despite high age-specific fertility rate and multiple dependency is a striking result that may be relatively unique to humans. Under natural fertility conditions, humans maintain a birth rate that is roughly double that of chimpanzees, our nearest primate relatives^46^. Extensive cooperation within and among families permits mothers to wean offspring earlier than expected given human body size. Whereas other primate mothers, including green monkeys and yellow baboons must either increase their work effort or resting time relative to feeding time to support the increased energetic costs of pregnancy and lactation^47^, human mothers decrease their active workloads and receive food and support from spouses, parents, in-laws, siblings, older children and other group members^48^. Unlike other mammals, human mothers offset the costs of gestation and lactation by increasing food intake and decreasing energy expenditure, rather than increasing fat mobilization or altering metabolism^49^. Most adult animals, especially in non-social species, rely on their own efforts for feeding and care, whereas humans depend on “pooled energy budgets” to subsidize rapid fertility and multiple dependent offspring simultaneously^50^. Family formation through pair bonding and reliable social exchange and support networks among multiple generations of kin were critical human adaptations that allowed for both higher fertility in humans and a longer post-reproductive lifespan. Thus, we speculate that the pace of reproduction is adjusted to maternal condition, rather than vice versa, and that energetic subsidies received from the social environment help increase fertility and offset the costs to maternal health and nutrition that mothers would otherwise bear if relying only on their own work efforts. Future research will focus on testing the moderating relationship that labor and resource subsidies should have on both the dynamics of maternal reproduction, and their effects on maternal health.

## Methods

### Study Population

The Tsimane are forager-horticulturalists (population ≈15,000) living in the Beni Department of the Bolivian Amazon, dispersed in 90+ villages ranging in size from 40–550 inhabitants. Their diet remains largely traditional, with more than 90% of calories coming from horticulture (plantains, manioc, rice), fishing, hunting, and gathering fruits. There is no running water, electricity, plumbing or public sanitation. Fertility among Tsimane is high, with little evidence of any sustained decline in recent years. The average Tsimane woman has 9.1 births over her reproductive lifetime^27^, and effective contraceptive use is rare and inconsistent. Average ± SD age at first birth is 18.1 ± 2.7 yrs, with average ± SD interbirth interval of 30.7 ± 10.6 months (4.8% < 18 mos, 16.7% between 18 and 24 mos)^45^. Mean ± SD weaning time was estimated at 19.2 ± 7.3 months based on recall, although median weaning time based on prospective study is longer (27.5 ± 1.2 months)^36^. Women commonly report breastfeeding up to and often after the onset of pregnancy.

We employ cross-sectional and longitudinal data collected from the Tsimane Health and Life History Project (www.unm.edu/~tsimane) over the period August 2002 to December 2012. A team of trained physicians, lab technicians and anthropologists visited 18 to 90 villages per wave, collecting systematic clinical evaluations of the entire census population each visit. Reproductive histories and other demographic information were first collected in 2002–2005^42^ and have since been updated in subsequent medical visits. Reproduction is measured by the number of live births (parity) and the pace of reproduction (1/average inter-birth interval, IBI). Anthropometric indices include weight (kgs) and body fat (%) as measured by bioelectric impedance using a Tanita scale, and body mass index (BMI) (kg/m^2^). Height was measured using a portable Seca 213 stadiometer. Women’s anthropometry was assessed on average 4.8 times (SD = 2.10, range = 1 to 9), spanning on average 7.13 years (SD = 2.24, range = 0.32 to 10.34). Descriptive statistics on the sample of women are given in Table S1.

During clinical evaluation, medical history, current symptoms and diagnoses, dental exams, hematology, fecal and urine analysis were assessed by project physicians. In-field blood analysis of fasting venous samples provided estimates of erythrocyte sedimentation rate (ESR) (Westergren Method). WBC count is a biomarker of immune activation and possibly infection, while ESR is a non-specific indicator of inflammation^26^. Leukocytosis we define as WBC > 10.0 × 10^9^ cells/L, and elevated ESR as ESR > 20 mm/hour (ages 15–44) or >30 mm/hour (ages 45+). Hemoglobin and WBC were measured with the QBC Autoread Plus dry hematology system (Drucker Diagnostics, State College, PA).

All methods and procedures were approved and conducted in accordance with guidelines set by the Institutional Review Boards of University of California, Santa Barbara (#12–496) and University of New Mexico (#07–157). Informed consent was given by the Tsimane Government (*Gran Consejo Tsimane*), village leaders, and all study participants directly.

### Analysis

Our initial analyses investigate sustained, long-term nutritional deficits by dividing women into three groups based on their longitudinal anthropometric trajectories: increasing, oscillating, and decreasing nutritional status (Table 1). Women were considered increasing if mean anthropometric trajectory increased by >0.3 standard deviations (SD). Women who were decreasing had trajectories decreasing by >0.3 SD. Women with oscillating trajectories had mean increases or decreases <0.3 SD. Multinomial logistic regression analyses are used to model whether parity and reproductive pace predict the likelihood of declining nutritional status. Other covariates include potential confounders (years of schooling, Spanish fluency [0 = none, 1 = some, 2 = fluent]) and controls (age, age at first birth, time since recent birth [in months], and pregnancy status).

A second, more fine-grained modeling approach takes advantage of the full anthropometric and demographic datasets and accounts for repeated measures on the same women. We use multilevel mixed-effects models to examine effects of parity and pace on changes in nutritional status, with random intercepts for each woman’s initial measure (e.g. weight) and random slopes allowing women’s rate of change to vary over time. Mixed effects models of biomarkers incorporated random intercepts but not slopes, due to fewer data points available per person. Models control for age at baseline measurement, Spanish fluency, schooling, pregnancy status, age at first birth, cohort and time elapsed since last birth. Models including the interactions between Spanish fluency, schooling and reproductive variables were included in the set of models compared, and final models were chosen based on AIC. Weighted Akaike scores (wAIC) are reported for each final model. Summaries of the alternative models in each set for Tables 3 and 4 are given in Tables S6 and S7, respectively. Additional models that only included women with at least 2 samples show very similar findings as those reported here, and are shown in Tables S8 and S9. All analyses were performed in STATA 13.

## Additional Information

**How to cite this article:** Gurven, M. *et al*. Health costs of reproduction are minimal despite high fertility, mortality and subsistence lifestyle. *Sci. Rep.* **6**, 30056; doi: 10.1038/srep30056 (2016).

## Supplementary Material

Supplementary Information

## Figures and Tables

**Table 1:** Categorization of longitudinal nutritional status trajectories of Tsimane women aged 20+ (n = 542).

	Weight		Body Fat		BMI	
	N	%	N	%	N	%
1. Declining	42	7.7	49	9.0	45	8.3
Sustained	13	2.4	10	1.8	18	3.3
Oscillate-High (−)	29	5.4	39	7.2	27	5.0
2. Increasing	155	28.6	143	26.4	153	28.2
Sustained	50	9.2	44	8.1	47	8.7
Oscillate-High (+)	105	19.4	99	18.3	106	19.6
3. Relatively Stable	338	62.4	345	63.7	336	62.0
Zero Mean	5	0.9	5	0.9	2	0.4
Oscillate-Slight (−)	128	23.6	127	23.4	134	24.7
Oscillate-Slight (+)	205	37.8	213	39.3	200	36.9

For declining status, sub-categorization includes sustained (unilateral consistent decline), and high (oscillation >0.3 SD of the individual mean, with mean decline >0.3 SD). Increasing status includes sustained (unilateral consistent increase) and high (oscillation >0.3 SD of the individual mean, with mean increase >0.3 SD). Relatively stable category includes slight oscillations within 0.3 SD of the individual mean, either declines (−) or increases (+), and those with no mean change.

**Table 2:** Predictors of declining anthropometric trajectory of women from logistic regression.

Predictors	Weight		Body Fat		BMI	
	RR	P>z	RR	P>z	RR	P>z
Mean Age (yrs)	1.060	0.217	*0.891*	0.057	1.076	0.137
Age at First Birth (yrs)	0.908	0.178	1.092	0.253	0.895	0.129
Months Since Last Birth	0.999	0.845	**1.011**	0.049	0.997	0.543
Mean Parity	0.944	0.598	1.103	0.464	0.882	0.287
Fertility Pace (1/Mean IBI)	1.104	0.901	0.990	0.990	1.617	0.690
Highest Grade	1.125	0.311	*0.742*	0.052	1.101	0.370
Spanish Fluency (0–2)	1.074	0.832	1.273	0.442	1.266	0.454
Pregnant (prop’n of obs)	**11.406**	0.027	3.399	0.213	**10.075**	0.027

Declining category refers to those with sustained or oscillate-high declines (see Table 1). Baseline includes all other categories. Bold indicates p < 0.05, italics p < 0.1. RR refers to Relative Risk.

**Table 3:** Mixed effects model of maternal weight, body fat and BMI for women age 15–44, and ages 45+.

Outcome	Weight				Body Fat				BMI			
Age	15–44		45+		15–44		45+		15–44		45+	
Predictor	β	P	β	P	β	P	β	P	β	P	β	P
Age (at Measurement) (yrs)	**1.050**	**0.000**	**1.222**	**0.001**	*0.451*	*0.052*	−0.161	0.680	**0.405**	**0.000**	**0.428**	**0.005**
Age*Age	−**0.007**	**0.013**	−**0.013**	**0.000**	0.001	0.708	0.001	0.656	−**0.002**	**0.032**	−**0.004**	**0.000**
Age at First Birth (yrs)	−**0.355**	**0.026**	*0.427*	*0.089*	−*0.305*	*0.055*	0.013	0.951	−**0.179**	**0.006**	0.105	0.305
Months Since Last Birth	−0.014	0.124	0.023	0.167	−0.005	0.657	−0.003	0.808	−**0.006**	0.090	0.006	0.411
Parity at Measurement	−**2.214**	**0.000**	1.431	0.226	−**1.861**	**0.008**	−0.095	0.922	−**0.985**	**0.000**	0.434	0.366
Pace of Fertility (1/IBI)	−**5.842**	**0.002**	2.005	0.595	−**5.271**	**0.008**	−0.253	0.934	−**2.815**	**0.000**	0.856	0.579
Mean Grade Completed	−0.118	0.509	1.328	0.108	−0.102	0.504	0.824	0.214	−0.103	0.140	0.075	0.816
Spanish Fluency (0–2)	**2.277**	**0.000**	**2.458**	**0.018**	**1.467**	**0.001**	**1.998**	**0.017**	**0.859**	**0.000**	**1.228**	**0.003**
Pregnancy Status (yes/no)												
Pregnant (vs. not)	**0.801**	0.001			−0.476	0.127			**0.324**	**0.002**		
Unknown (vs. not)	−0.316	0.231			−**1.036**	**0.003**			−0.179	0.119		
Season^a^	−**0.809**	0.000	−**1.006**	0.000	−**1.626**	**0.000**	−**1.433**	**0.000**	−**0.336**	**0.000**	−**0.440**	**0.000**
Time Period^b^	0.293	0.153	0.078	0.785	−0.256	0.322	**1.374**	**0.000**	0.087	0.324	−0.072	0.565
Parity*Pace	**3.037**	0.001	−1.950	0.277	**2.786**	**0.004**	0.025	0.986	**1.398**	**0.000**	−0.622	0.391
Intercept	**41.988**	0.000	7.546	0.464	**21.867**	**0.000**	**30.878**	**0.008**	**20.294**	**0.000**	*7.611*	*0.076*
**Random Effects**	**SD**	**SE**	**SD**	**SE**	**SD**	**SE**	**SD**	**SE**	**SD**	**SE**	**SD**	**SE**
Age	0.310	0.040	0.327	0.051	0.322	0.047	0.199	0.103	0.124	0.017	0.129	0.022
Intercept	9.045	1.229	18.717	2.874	10.592	1.382	10.815	6.176	3.489	0.540	6.480	1.331
Residual	3.045	0.059	2.413	0.081	4.147	0.078	4.066	0.197	1.352	0.026	1.083	0.036
Weighted Akaike	0.54		0.62		0.56		0.51		0.59		0.58	
N (Obs, Women)	2233, 592		853, 277		2212, 591		849, 277		2233, 592		853, 277	

Random effects terms are included for intercepts and slopes. Bold indicates p < 0.05, italics indicates p < 0.10.

^a^Season: 1 = Wet, 0 = Dry

^b^Time period: 1 = 2007–2013, 0 = 2002–2006.

**Table 4:** Mixed effects models of maternal hemoglobin (Hb), leukocyte count (WBC), and erythrocyte sedimentation rate (ESR) for women age 15–44, and ages 45+.

Outcome	Hb				WBC				Log(ESR)			
Age	15–44		45+		15–44		45+		15–44		45+	
Predictor	β	P	β	P	β	P	β	P	β	P	β	P
Age at Measurement (yrs)	0.024	0.482	0.010	0.736	−0.099	0.361	−**0.054**	**0.001**	−0.006	0.715	*0.006*	*0.061*
Age at First Birth (yrs)	0.022	0.564	−0.026	0.433	0.033	0.784	0.050	0.223	−0.012	0.534	0.010	0.309
Months since last birth	0.003	0.304	−0.002	0.509	−0.003	0.759			−0.002	0.243		
Parity at Measurement	−0.068	0.704	0.141	0.429	0.137	0.811	−0.354	0.344	0.040	0.658	−0.042	0.236
Pace of Fertility (1/IBI)	−0.184	0.697	−0.405	0.866	0.077	0.959	**15.022**	**0.025**	0.110	0.646	−**0.393**	**0.017**
Mean Grade Completed	−0.019	0.533	−**1.081**	**0.001**	−0.023	0.801	**0.438**	**0.035**	−0.020	0.211	0.030	0.556
Spanish Fluency (0–2)	0.118	0.188	0.136	0.293	−**1.092**	**0.000**	−0.670	0.378	0.007	0.889	−0.063	0.318
Pregnant (Yes vs. No) (Unknown vs. No)	−**0.694**	**0.000**			−0.361	0.274			**0.315**	**0.000**		
	−**0.345**	**0.022**			n/a	n/a			0.083	0.180		
Season^a^	−0.026	0.752	−**0.348**	**0.001**	−0.377	0.132	−0.215	0.435	*0.072*	*0.095*	**0.161**	**0.002**
Period^b^	0.153	0.150										
Parity*Pace	0.114	0.631	0.148	0.162	−0.144	0.850	0.381	0.241	−0.057	0.631	0.102	0.114
Body Fat %^1^	**0.028**	**0.000**	−0.027	0.908	−*0.029*	*0.100*	0.055	0.203	−0.001	0.875	−**0.011**	**0.006**
Body Fat*Pace^1^			**0.093**	**0.000**			−**0.367**	**0.018**				
Body Fat*Parity^1^			0.018	0.850			0.006	0.321				
Spanish*Pace			−**0.006**	**0.046**			*4.887*	*0.089*				
Spanish*Parity							−*0.216*	*0.052*				
Constant	**10.667**	**0.000**	**10.897**	**0.000**	**15.442**	**0.000**	**9.691**	**0.000**	**3.781**	**0.000**	**3.349**	**0.000**
**Random Effects**	SD	SE	SD	SE	SD	SE	SD	SE	SD	SE	SD	SE
Intercept	0.506	0.072	0.705	0.077	1.788	0.200	1.102	0.239	0.242	0.037	0.351	0.034
Residual	1.101	0.036	0.922	0.042	2.185	0.141	1.986	0.130	0.504	0.019	0.457	0.020
Weighted Akaike	0.76		0.47		0.46		0.32		0.36		0.72	
N (Obs, Women)	911, 461		494, 224		499, 382		316, 197		693, 389		486, 221	

ESR is logged to normalize distributions. Random effects terms are included for intercepts and slopes. Bold indicates p < 0.05, italics indicates p < 0.10. All models control for pregnancy status, season and time period (not shown).

^1^Weight used instead of Body Fat for WBC regressions.

^a^Season: 1 = Wet, 0 = Dry.

^b^Time period: 1 = 2007–2013, 0 = 2002–2006.
