# Supplementary material for: Health costs of reproduction are minimal despite high fertility, mortality and subsistence lifestyle
Source: Sci Rep. 2016 Jul 20;6:30056. doi: 10.1038/srep30056 (PMC4951795; doi:10.1038/srep30056)
Supplement: Supplementary Information [file srep30056-s1.pdf]

Supplementary Material for:

Health costs of reproduction are minimal despite high fertility, mortality and subsistence lifestyle

Michael Gurven[1,] *, Megan Costa[2], Ben Trumble[1], Jonathan Stieglitz[3], Bret Beheim[4], Daniel Eid Rodriguez[5], Paul L. Hooper[6], Hillard Kaplan[4]

[1]Department of Anthropology, University of California-Santa Barbara

[2]Population Studies Center, Graduate Group in Demography, University of Pennsylvania

[3]Institute for Advanced Study in Toulouse, France

[4]Department of Anthropology, University of New Mexico

[5]Department of Medicine, Universidad de San Simón, Cochabamba, Bolivia

[6]Department of Anthropology, Emory University

5 Supplemental Figures

9 Supplemental Tables

Figure S1. Longitudinal trajectories of body fat among women age 15+. Women with (a) sustained weight declines, or oscillations with mean decrease >0.3 SD; (b) sustained weight increases, or oscillations with mean increase >0.3 SD; (c) oscillations with mean increase or decrease <0.3 SD.

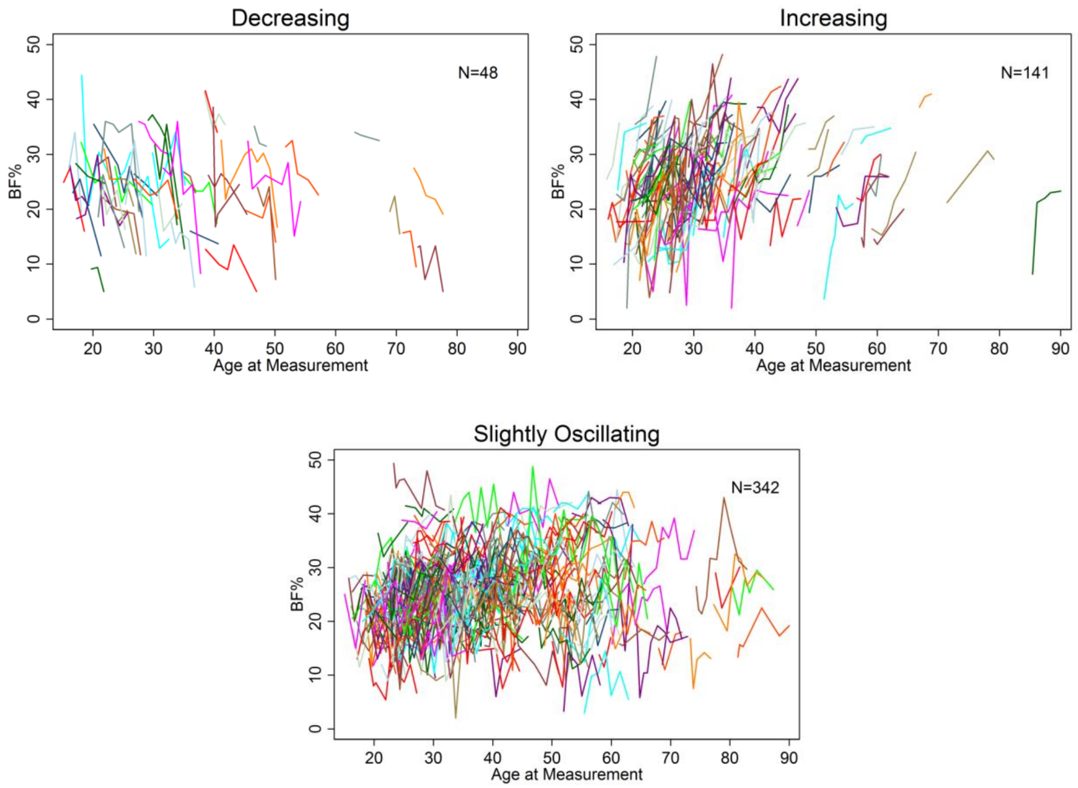

Figure S2. Longitudinal trajectories of body mass index (BMI) among women age 15+. Women with (a) sustained weight declines, or oscillations with mean decrease >0.3 SD; (b) sustained weight increases, or oscillations with mean increase >0.3 SD; (c) oscillations with mean increase or decrease <0.3 SD.

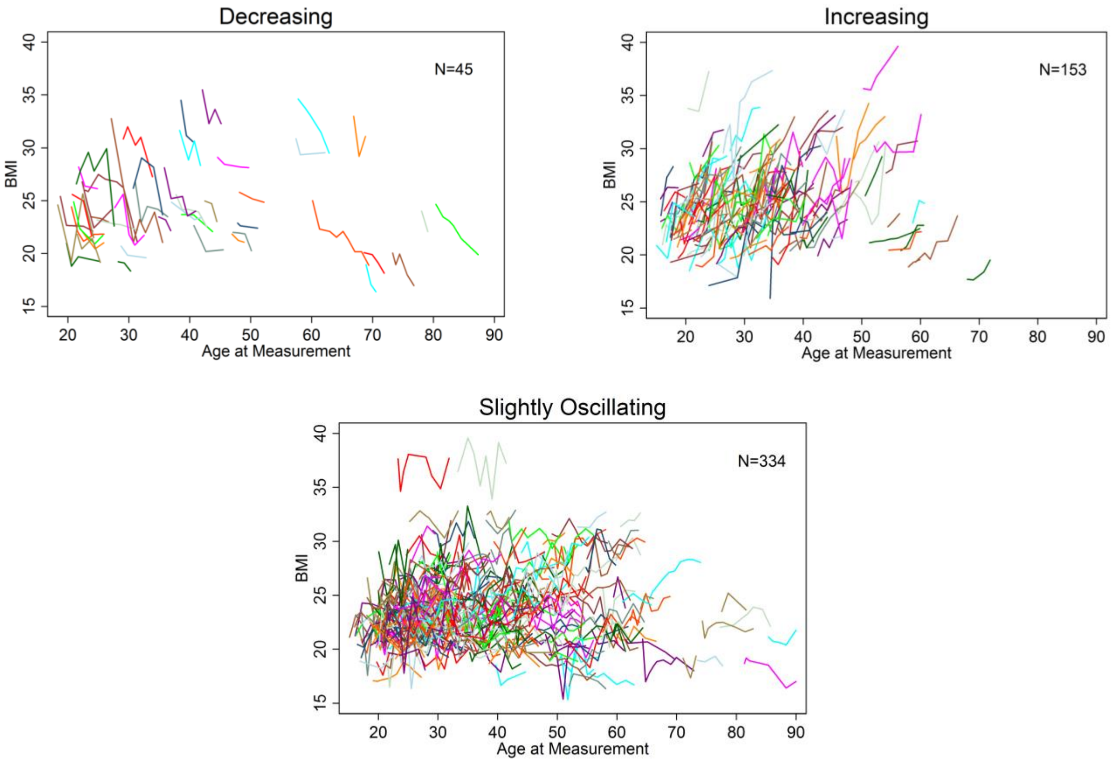

Figure S3. Predicted weight, body fat percentage and BMI, for women aged 15-44. Three lines represent reproductive trajectories varying in interbirth interval (IBI), mean ± 1 SD: short (1.67 yrs), mean (2.5 yrs) and long (3.33 yrs). Completed fertility is 13, 9 and 7 births, respectively. Predicted anthropometrics assume first birth at age 18, and then add the IBI to each age in the regression models given in Table 3, holding all other variables at sample average.

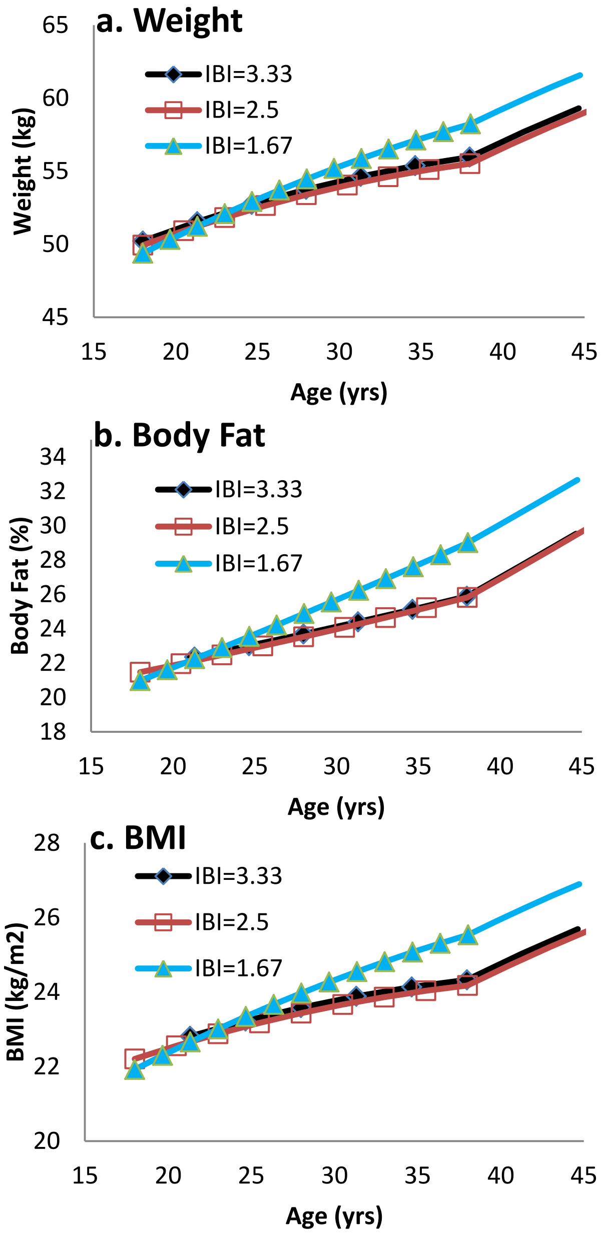

FIGURE S4. Longitudinal profiles of women's hemoglobin, white blood cell count and erythrocyte sedimentation rates. Black lines mark the clinical cutoffs for anemia (Hb<12 g/dL), leukocytosis (WBC>10.0 x 10^9 cells/L) and elevated ESR (>20 mm/hr for women age<50; >30 mm/hr age 50+).

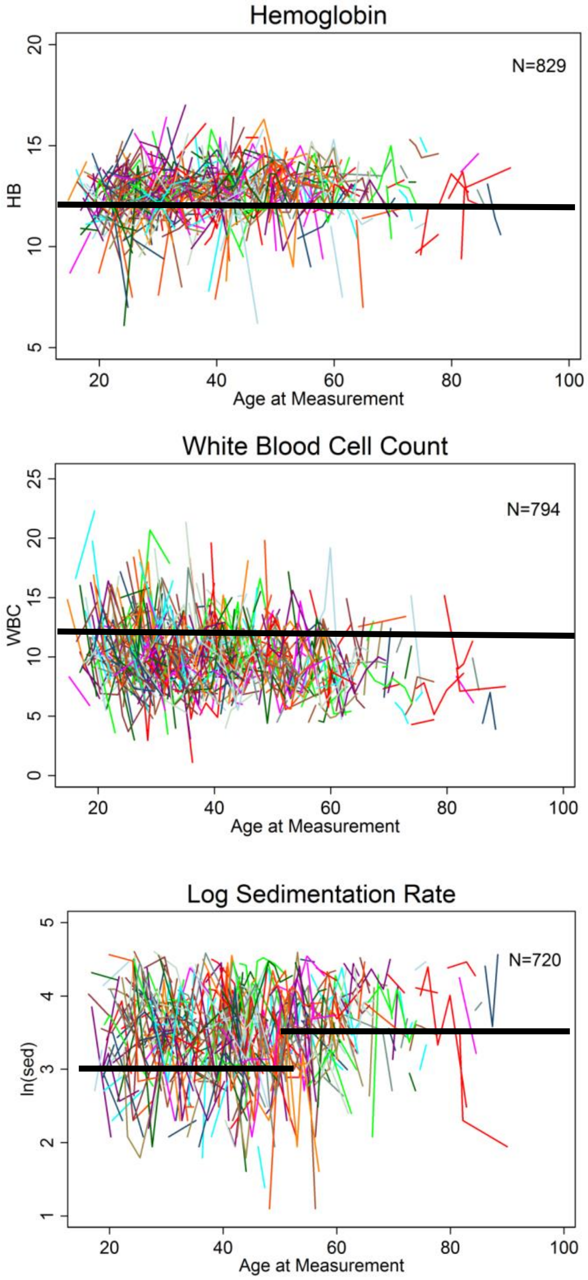

FIGURE S5. Predicted ESR in women age 45+ as a function of parity and IBI, based on model presented in Table 4. Control variables all held at sample average. Dashed blue line is the clinical cutoff for elevated ESR (30 mm/hr for women age 50+).

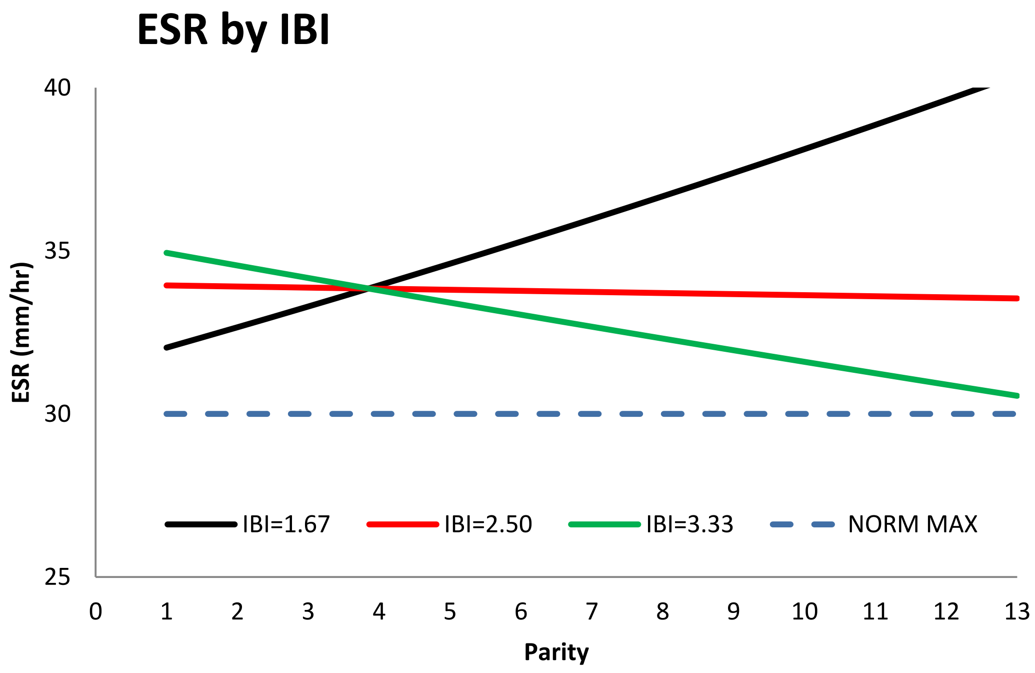

TABLE S1. Descriptive statistics for maternal measurements by age . Clinical cutoffs were used for anemia (Hb<12 g/dL), leukocytosis (WBC>10.0 x 10^9 cells/L), and elevated erythrocyte sedimentation rate (ESR>20 mm/hr) for women age 15-44, and ages 45+ (ESR>30 mm/hr).

	15-44			45+		
**Variable**	**Mean**	**SD**	**Obs, (N)**	**Mean**	**SD**	**Obs, (N)**
Age	30.65	7.46	2212, (591)	57.06	9.69	847, (277)
Age at First Birth	18.11	2.70	2212, (591)	19.49	4.11	847, (277)
Age at Last Birth	-	-	-	40.65	6.02	847, (277)
Months since recent birth	28.78	30.04	2212, (591)	207.11	133.86	847, (277)
Parity	5.41	2.94	2212, (591)	8.85	3.33	847, (277)
Pace (1/IBI)	0.45	0.52	2212, (591)	0.38	0.26	847, (277)
Highest Grade	1.54	2.03	2212, (591)	0.28	0.68	847, (277)
Spanish (0=none, 1=some, 2=fluent)	0.64	0.68	2212, (591)	0.38	0.56	847, (277)
Pregnant (1=yes)	0.15	0.35	1887, (576)	0.008	0.09	731, (271)
Season (1=wet, 0=dry)	0.52	0.50	2212, (591)	0.46	0.50	847, (277)
Period (1=2007-2013, 0=2002-2006)	0.62	0.48	2212, (591)	0.65	0.48	847, (277)
Height (cm)	150.89	4.37	2212, (591)	150.28	4.86	847, (277)
Weight (kg)	54.51	8.17	2212, (591)	53.91	9.90	847, (277)
BMI (kg/m^2)	23.90	3.19	2212, (591)	23.82	3.96	847, (277)
Overweight (BMI 25-29.9) (Prop’n)	0.26	0.43	2212, (591)	0.25	0.43	847, (277)
Obese (BMI≥30) (Prop’n)	0.05	0.21	2212, (591)	0.09	0.29	847, (277)
Body Fat (%)	24.98	7.14	2212, (591)	26.60	8.35	847, (277)
ESR (mm/hr)	37.66	20.20	693, (389)	40.04	21.11	486, (237)
Elevated ESR (Prop'n)	0.80	0.40	693, (389)	0.57	0.49	486, (237)
WBC (x10^9 cells/L)	10.61	2.09	496, (382)	9.49	2.43	316, (197)
Leukocytosis (Prop'n)	0.53	0.50	497, (382)	0.39	0.49	316, (197)
Hb (g/dL)	12.50	1.30	911, (461)	12.83	1.26	494, (224)
Anemia (Prop'n)	0.28	0.45	911, (461)	0.18	0.38	494, (224)

Table S2. Predictors of Declining Growth Trajectory. Declining category refers to those with sustained or oscillate-high declines (see Table 1). Baseline includes all other categories. Bold indicates p<0.05, italics p<0.1. Same as Table 2 but with interaction terms. RR refers to Relative Risk ratio.

	Weight		Body Fat		BMI	
**Predictors**	**RR**	**P>z**	**RR**	**P>z**	**RR**	**P>z**
Age at measurement	1.037	0.715	0.964	0.741	1.003	0.974
Age at first birth	0.924	0.502	1.005	0.968	0.962	0.740
Months since last birth	1.001	0.924	1.004	0.633	1.003	0.734
Mean Parity	1.155	0.797	0.692	0.529	1.443	0.517
Mean IBI inverse	1.716	0.756	0.278	0.470	6.408	0.373
Highest Grade	1.192	0.392	0.691	0.186	1.143	0.497
Spanish Fluency (0-2)	1.957	0.360	1.489	0.530	1.898	0.360
Pregnant (Prop'n of obs)	**11.517**	**0.027**	3.587	0.195	**9.950**	**0.028**
Spanish*Parity	0.924	0.419	0.979	0.823	0.946	0.574
Grade*Parity	0.969	0.475	1.015	0.772	0.980	0.616
Parity*IBI inverse	0.805	0.782	1.883	0.416	0.518	0.414

Table S3. Predictors of growth trajectories using multinomial regression. Declining category refers to those with sustained or oscillate-high declines (see Table 1). Increasing category refers to those with sustained increases or oscillate-high increases (see Table 1). Baseline includes slight oscillations (<0.3 SD). Models control for age at measurement, age at first birth, mean time since the recent birth, and pregnancy. Model 1 includes main effects for parity, fertility pace (1/IBI), schooling and Spanish fluency. Model 2 includes these main effects and interaction terms. RR refers to Relative Risk ratio.

	**Model 1**		**Model 2**		**Model 1**		**Model 2**	
**Predictors**	**RR**	**P>z**	**RR**	**P>z**	**RR**	**P>z**	**RR**	**P>z**
**WEIGHT**		**Increasing**				**Decreasing**		
Mean Parity	1.069	0.400	1.275	0.566	0.970	0.785	1.314	0.633
Mean 1/IBI	1.220	0.459	1.846	0.592	0.982	0.983	2.023	0.696
Highest Grade	1.038	0.596	0.825	0.187	1.114	0.372	1.205	0.378
Spanish Fluency	1.305	0.189	**2.643**	0.029	1.006	0.986	1.737	0.456
Spanish*Parity			*0.880*	0.073			0.930	0.454
Grade*Parity			*1.047*	0.087			0.964	0.411
Parity*1/IBI			0.806	0.701			0.698	0.654
**BODY FAT**		**Increasing**				**Decreasing**		
Mean Parity	1.044	0.591	0.979	0.957	1.121	0.403	0.734	0.601
Mean 1/IBI	1.299	0.502	1.045	0.970	0.906	0.914	0.289	0.493
Highest Grade	1.043	0.558	0.799	0.125	**0.726**	0.039	0.674	0.166
Spanish Fluency	0.954	0.825	1.949	0.148	1.260	0.466	1.500	0.526
Spanish*Parity			*0.878*	0.083			0.977	0.802
Grade*Parity			**1.058**	0.041			1.016	0.768
Parity*1/IBI			1.107	0.852			1.775	0.464
**BMI**		**Increasing**				**Decreasing**		
Mean Parity	1.024	0.768	1.515	0.317	0.922	0.443	1.699	0.362
Mean 1/IBI	1.377	0.539	3.871	0.278	1.176	0.816	6.220	0.312
Highest Grade	1.031	0.659	0.915	0.527	1.089	0.444	1.164	0.451
Spanish Fluency	1.373	0.116	*2.147*	0.083	1.168	0.626	1.589	0.511
Spanish*Parity			0.922	0.236			0.977	0.802
Grade*Parity			1.023	0.377			1.016	0.768
Parity*1/IBI			0.596	0.352			1.775	0.464

Table S4. Mixed effects model of maternal Anemia (Hb<12 g/dL), leukocytosis (WBC>10.0 x 10^9 cells/L), and elevated erythrocyte sedimentation rate (ESR>20 mm/hr) for women age 15-44, and ages 45+ (ESR>30 mm/hr). Random effects terms are included for intercepts and slopes. Bold indicates p<0.05, italics indicates p<0.10. All models control for pregnancy status, season and time period (not shown). OR refers to Odds Ratio.

Outcome:	Anemia				Leukocytosis				Elevated ESR			
Age:	15-44		45+		15-44		45+		15-44		45+	
**Predictor**	OR	P	OR	P	OR	P	OR	P	OR	P	OR	P
Age at Measurement	0.810	0.116	0.851	0.401	0.883	0.333	0.947	0.483	0.904	0.322	*1.029*	*0.099*
Age*Age	**1.004**	**0.039**	1.000	0.759								
Age at First Birth	0.899	0.220	1.184	0.207	1.014	0.922	1.000	0.998	1.004	0.972	*1.086*	*0.083*
Months Since Last Birth	**0.986**	**0.046**	1.009	0.347	1.002	0.875	1.001	0.856	1.000	0.954		
Parity at Measurement	0.799	0.598	1.514	0.435	1.100	0.882	1.108	0.800	1.464	0.493	0.827	0.314
Pace of Fertility (1/IBI)	0.115	0.460	0.004	0.279	0.109	0.340	0.588	0.834	0.014	0.293	0.066	0.426
Mean Grade Completed	1.055	0.435	**1.640**	**0.043**	1.062	0.560	*1.469*	0.093	0.963	0.657	1.157	0.556
Spanish Ability	2.514	0.148	1.307	0.389	**0.250**	**<0.001**	**0.487**	0.028	0.776	0.330	0.913	0.765
Pregnant (yes vs. no)	**4.786**	**<0.001**			0.696	0.335			*2.605*	*0.055*		
(unknown vs. no)	**2.286**	**0.011**							1.005	0.990		
Wet Season (1=wet)	1.111	0.566	1.374	0.306	*0.609*	0.082	1.020	0.948	1.179	0.498	**1.881**	**0.024**
Period 2007-2013	0.747	0.219	*0.558*	0.064								
Parity*Pace	1.781	0.274	0.832	0.771	1.401	0.691	0.882	0.824	0.535	0.361	1.609	0.260
Body Fat %	0.968	0.444	**0.900**	**<0.001**					0.910	0.111	**0.941**	**0.003**
Weight					*0.965*	*0.076*						
Body Fat*Pace	1.075	0.374							1.190	0.135		
Body Fat*Parity	0.993	0.117							1.006	0.313		
Spanish*Pace	**0.063**	**0.021**										
Spanish*Parity	0.998	0.969										
**Random Effects**	SD	SE	SD	SE	SD	SE	SD	SE	SD	SE	SD	SE
Intercept	0.816	0.225	1.123	0.353	1.697	0.424	0.882	0.391	1.257	0.324	1.436	0.279
Weighted Akaike	0.49		0.67		0.66		0.42		0.29		0.79	
N (Obs, Women)	911, 461		494, 224		499,382		313, 195		693, 389		486, 221	

[1]Weight used instead of Body Fat for WBC regression

Table S5. Comparison of nulliparous and parous women age 45+.

	45+, Nulliparous			45+, min 1 live birth			
	**Mean**	**SD**	**Obs, (N)**	**Mean**	**SD**	**Obs, (N)**	**p-value**
Age	64.46	12.04	35, (9)	57.06	9.69	847, (277)	0.112
Height (cm)	146.95	4.97	31, (8)	150.28	4.86	847, (277)	0.088
Weight (kg)	46.85	6.34	31, (8)	53.91	9.9	847, (277)	0.016
BMI (kg/m^2)	21.70	2.68	31, (8)	23.82	3.96	847, (277)	0.061
Body Fat (%)	22.85	6.27	30, (8)	26.6	8.35	847, (277)	0.132
ESR (mm/hr)	49.35	19.97	20, (7)	40.04	21.11	486, (237)	0.285
Elevated ESR (Prop'n)	0.80	0.41	20, (7)	0.57	0.49	486, (237)	0.226
WBC ($x10^9$ cells/L)	8.52	2.71	9, (7)	9.49	2.43	316, (197)	0.399
Leukocytosis (Prop'n)	0.11	0.33	9, (7)	0.39	0.49	316, (197)	0.134
Hb (g/dL)	11.83	1.26	20, (7)	12.83	1.26	494, (224)	0.092
Anemia (Prop'n)	0.55	0.51	20, (7)	0.18	0.38	494, (224)	0.029

Note: p-value is from two-sample t-test, but given the small n for nulliparous women, it is only shown for illustration.

Table S6. Summary of model selection leading to best-fit models presented in Table 3. "+" signifies positive coefficient, "-" negative coefficient. Double symbol reflects p<0.05, large/bolded reflects 0.05<p<0.1 and small/unbolded p>0.1. Grey shading highlights best-fit model reported in Table 3.

Model No.	Age	Age^2	Age at first birth	Months since last birth	Parity	Pace (1/IBI)	Mean Grade	Spanish Fluency	Pregnant	Season	Time Period	Parity*Pace	Spanish*Pace	Spanish*Parity	Grade*Pace	Grade*Parity	AIC	Δ_i	w_i
**Weight, Ages 15-44**																			
1	++	– –	– –	-	– –	– –	-	++	++	– –	+	++					13113.56	0	0.54
2	++	– –	– –	-	– –	**–**	-	++	++	– –	+	++	**–**	-			13114.34	0.78	0.37
3	++	– –	– –	-	– –	– –	-	++	++	– –	+	++			-	-	13117.14	3.58	0.09
**Weight, Ages 45+**																			
1	++	– –	**+**	+	+	+	+	++		– –	+	-					4979.64	0	0.62
2	++	– –	+	+	+	+	+	-		– –	+	-	+	-			4982.22	2.58	0.17
3	++	– –	+	+	+	+	+	++		– –	+	-			+	+	4981.88	2.24	0.20
**Body Fat, Ages 15-44**																			
1	++	+	– –	-	– –	– –	-	++	-	– –	-	++					13760.69	0	0.56
2	++	+	**–**	– –	– –	-	-	++	-	– –	-	++	-	-			13761.61	0.92	0.35
3	+	+	**–**	-	– –	– –	+	++	-	– –	-	++			-	-	13764.27	3.58	0.09
**Body Fat, Ages 45+**																			
1	-	+	+	-	-	-	+	+		-	++	+					5392.38	0	0.51
2	-	+	+	-	-	-	+	+		-	++	+	**+**	-			5392.8	0.42	0.42
3	-	+	+	-	-	-	+	+		-	++	+			-	+	5396.28	3.9	0.07
**BMI, Ages 15-44**																			
1	++	– –	– –	**–**	– –	– –	-	++	++	– –	+	++					9321.18	0	0.59
2	++	– –	– –	**–**	– –	– –	**–**	++	++	– –	+	++	-	-			9322.42	1.24	0.32
3	++	– –	– –	**–**	– –	– –	-	++	++	– –	+	++			-	-	9324.89	3.71	0.09
**BMI, Ages 45+**																			
1	++	– –	+	+	+	+	+	++		– –	-	-					3544.52	0	0.58
2	++	– –	+	+	+	+	+	+		– –	-	-	+	-			3546.13	1.61	0.26
3	++	– –	+	+	+	+	+	++		– –	-	-			+	+	3547.04	2.52	0.16

Table S7. Summary of model selection leading to best-fit models presented in Table 4. “+” signifies positive coefficient, “-“ negative coefficient. Double symbol reflects p<0.05, large/bolded reflects 0.05<p<0.1 and small/unbolded p>0.1. Grey shading highlights best-fit model reported in Table 4.

Model No.	Age	Age^2	Age at first birth	Months since last birth	Parity	Pace (1/IBI)	Mean Grade	Spanish Fluency	Pregnant	Season	Period	Parity*Pace	Weight /Body Fat	Body Fat*Pace	Body Fat*Parity	Spanish*Pace	Spanish*Parity	Grade*Pace	Grade*Parity	AIC	Δ_i	w_i
**Hemoglobin (Hb), Ages 15-44**																						
1	**++**	**++**	+	+	-	-	-	**+**	**– –**	-	+	+								2987.35	39.35	0.00
2	+		+	+	-	-	-	+	**– –**	-	+	+	**++**							2948	0	0.76
3	**+**	**–**	+	+	-	-	-	+	**– –**	-	+	+	**++**	-	-					2951.43	3.43	0.14
4	**+**	**–**	+	+	-	-	-	-	**– –**	-	+	+	**++**	-	-	+	+			2952.64	4.64	0.07
5	**+**	**–**	+	+	-	-	-	+	**– –**	-	+	+	**++**	-	-			+	+	2954.37	6.37	0.03
**Hemoglobin (Hb), Ages 45+**																						
1	+		-	-	-	-	**–**	+		**– –**	**++**	+								1578.4	46.99	0.00
2	-		-	-	-	-	**– –**	+		**– –**	+	+	**++**							1536.7	5.29	0.03
3	+		-	-	+	-	**– –**	+		**– –**	+	+	**++**	+	**– –**					1535.4	3.99	0.06
4	+		-	-	+	-	**– –**	**– –**		**– –**	+	+	**++**	+	**– –**	**+**	+			1531.59	0.18	0.43
5	+		-	-	+	-	**– –**	+		**– –**	+	+	**++**	+	**– –**			+	+	1531.41	0	0.47
**White Blood Cells (WBC), Ages 15-44**																						
1	-		+	-	+	+	-	**– –**	-	-		-								2459.04	0.71	0.33
2	-		+	-	+	+	-	**– –**	-	-		-	**–**							2458.33	0	0.46
3	-		+	-	+	-	-	**– –**	-	-		-	-	+	-					2462.04	3.71	0.07
4	-		+	-	+	-	-	**– –**	-	-		-	-	+	-	+	+			2464.24	5.91	0.02
5	-		+	-	-	-	**–**	**– –**	-	-		-	-	+	-			**++**	+	2461.15	2.82	0.11
**White Blood Cells (WBC), Ages 45+**																						
1	**– –**		+		-	-	**+**	**– –**		-		+								1448.81	9.58	0.00
2	**– –**		+		-	-	**++**	**– –**		-		+	**–**							1439.53	0.3	0.27
3	**– –**		+		-	+	**++**	**– –**		-		+	+	**–**	+					1439.23	0	0.32
4	**– –**		+		-	**++**	**++**	-		-		+	+	**– –**	+	**+**	**–**			1439.33	0.1	0.30
5	**– –**		+		-	**+**	-	**– –**		-		+	+	**– –**	+			+	-	1441.51	2.28	0.10
**Ln(Erythrocyte Sedimentation Rate), Ages 15-44**																						
1	-		-	-	+	+	-	+	**++**	**+**		-								1176.05	0.33	0.31
2	-		-	-	+	+	-	+	**++**	**+**		-	-							1175.72	0	0.36
3	-		-	-	+	+	-	+	**++**	**+**		-	-	+	-					1177.59	1.87	0.14
4	-		-	-	+	+	-	+	**++**	**+**		-	-	+	-	-	+			1179.19	3.47	0.06
5	-		-	-	+	+	**–**	+	**++**	**+**		-	-	+	-			+	**+**	1177.89	2.17	0.12
**Ln(Erythrocyte Sedimentation Rate), Ages 45+**																						
1	**++**		+		-	**– –**	+	-		**++**		**+**								821.24	7.39	0.02
2	**+**		+		-	**– –**	+	-		**++**		+	**– –**							813.85	0	0.72
3	**+**		+		-	+	+	-		**++**		**+**	-	-	+					816.89	3.04	0.16
4	**+**		+		-	+	+	+		**++**		**+**	-	-	+	-	-			820.61	6.76	0.02
5	**+**		+		-	-	+	-		**++**		**++**	-	-	+			-	+	818.42	4.57	0.07

Table S8. Mixed effects model of maternal weight, body fat and BMI for women age 15-44, comparing sample of women with 1 or more versus 2 or more observations. Random effects terms are included for intercepts and slopes.

	Weight				Body Fat				BMI			
# Observations:	1+		2+		1+		2+		1+		2+	
**Predictor**	β	P	β	P	β	P	β	P	β	P	β	P
Age (at Measurement)	**1.050**	**<0.001**	**1.100**	**<0.001**	*0.451*	*0.052*	**0.496**	**0.041**	**0.405**	**<0.001**	**0.411**	**<0.001**
Age*Age	**-0.007**	**0.013**	**-0.007**	**0.014**	0.001	0.708	0.001	0.787	**-0.002**	**0.032**	*-0.002*	*0.055*
Age at First Birth	**-0.355**	**0.026**	*-0.337*	*0.051*	*-0.305*	*0.055*	*-0.284*	*0.098*	**-0.179**	**0.006**	**-0.178**	**0.012**
Months Since Last Birth	-0.014	0.124	*-0.015*	*0.095*	-0.005	0.657	-0.005	0.646	*-0.006*	*0.090*	*-0.007*	*0.075*
Parity at Measurement	**-2.214**	**<0.001**	**-2.252**	**0.001**	**-1.861**	**0.008**	**-1.837**	**0.013**	**-0.985**	**<0.001**	**-1.010**	**<0.001**
Pace of Fertility (1/IBI)	**-5.842**	**0.002**	**-5.779**	**0.003**	**-5.271**	**0.008**	**-4.987**	**0.017**	**-2.815**	**<0.001**	**-2.787**	**0.001**
Mean Grade Completed	-0.118	0.509	-0.320	0.130	-0.102	0.504	-0.211	0.219	-0.103	0.140	*-0.147*	*0.071*
Spanish Fluency (0-2)	**2.277**	**<0.001**	**3.249**	**<0.001**	**1.467**	**0.001**	**2.160**	**<0.001**	**0.859**	**<0.001**	**1.202**	**<0.001**
Pregnancy Status												
Pregnant (Baseline No)	**0.801**	**0.001**	**0.781**	**0.001**	-0.476	0.127	*-0.536*	*0.088*	**0.324**	**0.002**	**0.310**	**0.003**
Unknown	-0.316	0.231	-0.273	0.306	**-1.036**	**0.003**	**-1.079**	**0.002**	-0.179	0.119	-0.173	0.136
Season	**-0.809**	**<0.001**	**-0.798**	**<0.001**	**-1.626**	**<0.001**	**-1.645**	**<0.001**	**-0.336**	**<0.001**	**-0.329**	**<0.001**
Time Period	0.293	0.153	0.239	0.252	-0.256	0.322	-0.287	0.272	0.087	0.324	0.069	0.442
Parity*Pace	**3.037**	**0.001**	**2.975**	**0.002**	**2.786**	**0.004**	**2.635**	**0.010**	**1.398**	**<0.001**	**1.389**	**0.001**
Constant	**41.988**	**<0.001**	**40.331**	**<0.001**	**21.867**	**<0.001**	**20.188**	**<0.001**	**20.294**	**<0.001**	**19.917**	**<0.001**
**Random Effects**	**SD**	**SE**	**SD**	**SE**	**SD**	**SE**	**SD**	**SE**	**SD**	**SE**	**SD**	**SE**
ID(Age)	0.310	0.040	0.326	0.040	0.322	0.047	0.332	0.048	0.124	0.017	0.133	0.017
Intercept	9.045	1.229	9.528	1.252	10.592	1.382	10.617	1.413	3.489	0.540	3.856	0.539
Residual	3.045	0.059	3.038	0.059	4.147	0.078	4.139	0.078	1.352	0.026	1.346	0.026
N (Obs, Women)	2233, 592		2121, 480		2212, 591		2101, 480		2233, 592		2121, 480	

Table S9. Mixed effects model of maternal weight, body fat and BMI for women age 45+, comparing sample of women with 1 or more versus 2 or more observations. Random effects terms are included for intercepts and slopes.

	Weight				Body Fat				BMI			
# Observations:	1+		2+		1+		2+		1+		2+	
**Predictor**	β	P	β	P	β	P	β	P	β	P	β	P
Age (at Measurement)	**1.222**	**0.001**	**1.230**	**0.024**	-0.161	0.680	-0.044	0.935	**0.428**	**0.005**	-0.044	0.935
Age*Age	**-0.013**	**<0.001**	**-0.013**	**<0.001**	0.001	0.656	0.003	0.371	**-0.004**	**<0.001**	0.003	0.371
Age at First Birth	*0.427*	*0.089*	0.340	0.496	0.013	0.951	-0.332	0.435	0.105	0.305	-0.332	0.435
Months Since Last Birth	0.023	0.167	0.023	0.520	-0.003	0.808	-0.029	0.366	0.006	0.411	-0.029	0.366
Parity at Measurement	1.431	0.226	1.205	0.541	-0.095	0.922	-1.417	0.394	0.434	0.366	-1.417	0.394
Pace of Fertility (1/IBI)	2.005	0.595	-4.085	0.854	-0.253	0.934	5.433	0.764	0.856	0.579	5.433	0.764
Mean Grade Completed	1.328	0.108	0.896	0.369	0.824	0.214	0.398	0.610	0.075	0.816	0.398	0.610
Spanish Fluency (0-2)	**2.458**	**0.018**	**3.162**	**0.031**	**1.998**	**0.017**	**2.495**	**0.028**	**1.228**	**0.003**	**2.495**	**0.028**
Season	**-1.006**	**<0.001**	**-1.009**	**<0.001**	**-1.433**	**<0.001**	**-1.446**	**<0.001**	**-0.440**	**<0.001**	**-1.446**	**<0.001**
Time Period	0.078	0.785	0.014	0.962	**1.374**	**<0.001**	**1.397**	**0.001**	-0.072	0.565	**1.397**	**0.001**
Parity*Pace	-1.950	0.277	-1.473	0.551	0.025	0.986	1.294	0.512	-0.622	0.391	1.294	0.512
Constant	7.546	0.464	11.500	0.381	**30.878**	**0.008**	**36.054**	**0.009**	*7.611*	*0.076*	**36.054**	**0.009**
**Random Effects**	**SD**	**SE**	**SD**	**SE**	**SD**	**SE**	**SD**	**SE**	**SD**	**SE**	**SD**	**SE**
ID(age)	0.327	0.051	0.355	0.053	0.199	0.103	0.228	0.103	0.129	0.022	0.228	0.103
Intercept	18.717	2.874	20.010	3.058	10.815	6.176	12.191	6.192	6.480	1.331	12.191	6.192
Residual	2.413	0.081	2.397	0.081	4.066	0.197	4.063	0.133	1.083	0.036	4.063	0.133
N (Obs, Women)	853, 277		782, 206		849, 277		778, 206		853, 277		778, 206	
